# Ghrelin is involved in regulating the progression of *Echinococcus Granulosus-*infected liver lesions through suppression of immunoinflammation and fibrosis

**DOI:** 10.1371/journal.pntd.0012587

**Published:** 2024-10-22

**Authors:** Jiang Zhu, Hongqiong Zhao, Aili Aierken, Tanfang Zhou, Meng Menggen, Huijing Gao, Rongdong He, Kalibixiati Aimulajiang, Hao Wen

**Affiliations:** 1 State Key Laboratory of Pathogenesis, Prevention and Treatment of High Incidence Diseases in Central Asia, Clinical Medicine Institute, The First Affiliated Hospital of Xinjiang Medical University, Xinjiang, China; 2 Department of Hepatobiliary and Hydatid Disease, Digestive and Vascular Surgery Center Therapy Center, The First Affiliated Hospital of Xinjiang Medical University, Xinjiang, China; 3 College of Veterinary Medicine, Xinjiang Agricultural University, Xinjiang, China; Broad Institute Harvard: Broad Institute, UNITED STATES OF AMERICA

## Abstract

**Background:**

Cystic *Echinococcosis* (CE) is a zoonotic disease causing fibrosis and necrosis of diseased livers caused by infection with *Echinococcus granulosus* (*E*.*g*). There is evidence that *E*.*g* is susceptible to immune escape and tolerance when host expression of immunoinflammation and fibrosis is suppressed, accelerating the progression of CE. Ghrelin has the effect of suppressing immunoinflammation and fibrosis, and whether it is involved in regulating the progression of *E*.*g*-infected liver lesions is not clear.

**Methods:**

Serum and hepatic Ghrelin levels were observed in *E*.*g*-infected mice (4, 12 and 36 weeks) and compared with healthy control groups. Co-localization analysis is performed between protein expression of Ghrelin in and around the hepatic lesions of *E*.*g*-infected 12-week mice and protein expression of different hepatic histiocytes by mIHC. HepG2 cells and protoscoleces (PSCs) protein were co-cultured in vitro, as well as PSCs were alone in vitro, followed by exogenously administered of Ghrelin and its receptor blocker, [D-Lys3]-GHRP-6, to assess their regulatory effects on immunoinflammation, fibrosis and survival rate of PSCs.

**Results:**

Serum Ghrelin levels were increased in *E*.*g*-infected 4- and 12-week mice, and reduced in 36-week mice. *E*.*g*-infected mice consistently recruited Ghrelin in and around the hepatic lesions, which was extremely strongly co-localized with the protein expression of hepatic stellate cells (HSCs), T cells and the TGF-β1/Smad3 pathway. The secretion of Ghrelin was increased with increasing concentrations of PSCs protein in HepG2 cells culture medium. Moreover, Ghrelin could significantly inhibit the secretion of IL-2, INF-γ and TNF-α, as well as the expression of Myd88/NF-κB and TGF-β1/Smad3 pathway protein, and promoted the secretion of IL-4 and IL-10. Blocking Ghrelin receptor could significantly inhibit PSCs growth in *in vitro* experiment.

**Conclusion:**

Ghrelin is highly expressed in the early stages of hepatic *E*.*g* infection and may be involved in regulating the progression of liver lesions by suppression immunoinflammation and fibrosis.

## Author summary

There is evidence that *Echinococcus granulosus* (*E*.*g*) is susceptible to immune escape and tolerance when host expression of immunoinflammation and fibrosis is suppressed, accelerating the progression of hepatic cystic *Echinococcosis* (CE). Ghrelin has the effect of suppressing immunoinflammation and fibrosis, and whether it is involved in the regulation of CE progression is not clear. The present study demonstrated that Ghrelin is highly expressed in the early stage of hepatic *E*.*g* infection and may act mainly on hepatic stellate cells and T cells, exerting biological effects to inhibit Th1-type cellular immunity, the Myd88/NF-κB and TGF-β1/Smad3 pathways and agonize Th2-type cellular immunity, which in turn suppresses host expression of immunoinflammation and fibrosis. However, this biological effect of Ghrelin may regulate the progression of CE. [D-Lys3]-GHRP-6, as a Ghrelin receptor blocker, is expected to be a drug target against CE. In addition, we found protoscoleces of *E*.*g* with gene expression of Ghrelin, which also provides some evidence that Ghrelin regulates the disease progression of CE. Our study reveals the possible regulatory role and mechanism of Ghrelin in the process of hepatic *E*.*g* infection. In-depth studies could help to further unravel the disease mechanisms of CE and provide potential targets for therapy.

## Introduction

Cystic *Echinococcosis* (CE) is a chronic parasitic infectious liver disease caused by *Echinococcus granulosus* (*E*.*g*) infection that can lead to fibrosis and necrosis of the diseased liver. It is still an unresolved and serious global public health problem, with high prevalence in pastoral areas of Northwest China, the Middle East, North Africa and South America [1,2]. As symptoms of CE appear in the late stage of the disease, palliative surgery is often ineffective, leading to frequent recurrence of the disease [3,4]. The cure rate of albendazole, currently the world’s most recognized CE therapeutic drugs, is only about 30%, and there are serious complications of liver and kidney damage [5]. Therefore, it is necessary to continue to carry out research on the pathogenic mechanism of CE and to search for more effective immunotherapeutic targets or small-molecule compounds against CE.

Previous studies had reported that the imbalance of Th1/Th2-type cellular immunity in patients with CE is an important factor contributing to the development of *E*.*g* immune tolerance and escape, leading to disease progression. In experiments with *E*.*g*-infected mice, Th1-type cellular immunity predominated in the early stage of infection, and Th2-type cellular immunity predominated in the chronic stage of infection [6–12]. Moreover, the maintenance of a high response of Th1-type cellular immunity attenuates liver damage by *E*.*g* infection, while the maintenance of a high response of Th2-type cellular immunity facilitates the parasitism and survival of *E*.*g* on the host [13–18]. Clinical studies have concluded the same that high expression of Th1-type cellular immunity occurred in patients with initial infection and inactive CE populations [19–21] and high expression of Th2-type cellular immunity occurred in patients with recurrent infection and active CE compared to healthy populations [21–24]. In addition, effective anti-infection therapy for CE is positively correlated with the maintenance of high responses in Th1-type cellular immunity [13,25]. The NF-κB [19,26] and TGF-β1/Smad3 [20,27–29] signaling pathways likewise play key roles in regulating the expression of immunoinflammation and fibrosis in *E*.*g*-infected hosts. Inhibition of these pathways could attenuate host protective immunity and promote disease progression. Previous studies had shown that initial *E*.*g* infection activates the host NF-κB and TGF-β1/Smad3 signaling pathways, increases secretion of the pro-inflammatory factors IL-1β, IL-2, INF-γ and TNF-α, and activates hepatic stellate cells (HSCs) to mediate secretion of the pro-fibrotic factors fibronectin, α-SMA, collagen I and III secretion, and recruits histiocytes in and around liver lesions, including HSCs, T cells, macrophages and fibroblasts/myofibroblasts, to exert protective effects against *E*.*g* infection [19,30–34]. However, inhibition of the NF-κB [19,20,27,28] and TGF-β1/Smad3 [35,36] signaling pathways, combined with Th1-type cellular immunosuppression, could jointly promote disease progression in chronically infected and relapsed CE patients.

Ghrelin is an endogenous ligand of GHSR found in the human stomach in 1999, and the fundus of the stomach is its main secretory region. It is secreted by X/A-like cells in rodents and P/D1 cells in humans, with a high degree of homology [37]. Ghrelin is also widely underexpressed in central systems such as the hypothalamus, pituitary, cerebral cortex and striatum, as well as peripheral organs such as the liver, pancreas, gastrointestinal tract, heart, adrenal glands and ovaries [38]. It had been shown that Ghrelin is able to inhibit Th1-type cellular immunity [39–43], the NF-κB [44–47] and TGF-β1/Smad3 [45,48] signaling pathways after binding to the receptor GHSR, exerting a protective effect in ameliorating chronic inflammation and fibrosis formation in many benign liver diseases, including hepatitis, hepatic fibrosis, and cirrhosis [49]. However, as mentioned above, this effect of Ghrelin may promote *E*.*g* parasitism and survival on the host of CE patients. In addition, Ghrelin is able to regulate hepatic IGF-1 secretion via the "gastrointestinal-brain-hepatic axis" [49–51]. IGF-I could directly inhibit the proliferation and activation of HSCs to improve the progression of hepatic fibrosis [52–54], and parasite-related experiments had reported that IGF-1 promotes parasitism and survival of parasites on hosts, and accelerated disease progression [55–59]. Therefore, it is possible that Ghrelin is involved in regulating the progression of *E*.*g*-infected liver lesions in CE patients.

In the present study, we observed the changes in serum levels and hepatic protein expression of Ghrelin at different stages of *E*.*g*-infected mice, as well as co-localized with different hepatic histiocytes Furthermore, we also intervened with Ghrelin protein and its receptor blocker [D-Lys3]-GHRP-6 in *in vitro* co-cultures of HepG2 cells and protoscoleces (PSCs) proteins, as well as in *in vitro* cultures of PSCs alone, and evaluated their regulatory effects on immunoinflammation, fibrotic expression, and growth of PSCs, to further investigate whether Ghrelin has a regulatory role in the progression of hepatic *E*.*g* infection.

## Materials and methods

### Animals and ethical statement

All female BALB/c mice (6 weeks old and body mass 18~20 g) were purchased from Xinjiang Medical University, China, and were housed in a barrier environment at the Animal Experimentation Centre of Xinjiang Medical University, China. The study was approved by the Animal Ethics Committee of the First Affiliated Hospital of Xinjiang Medical University (Ethics Approval No.: IACUC-20230321014) and was conducted according to the guidelines of the Ethics Committee.

### PSCs isolation and culture

**Fig 1:**
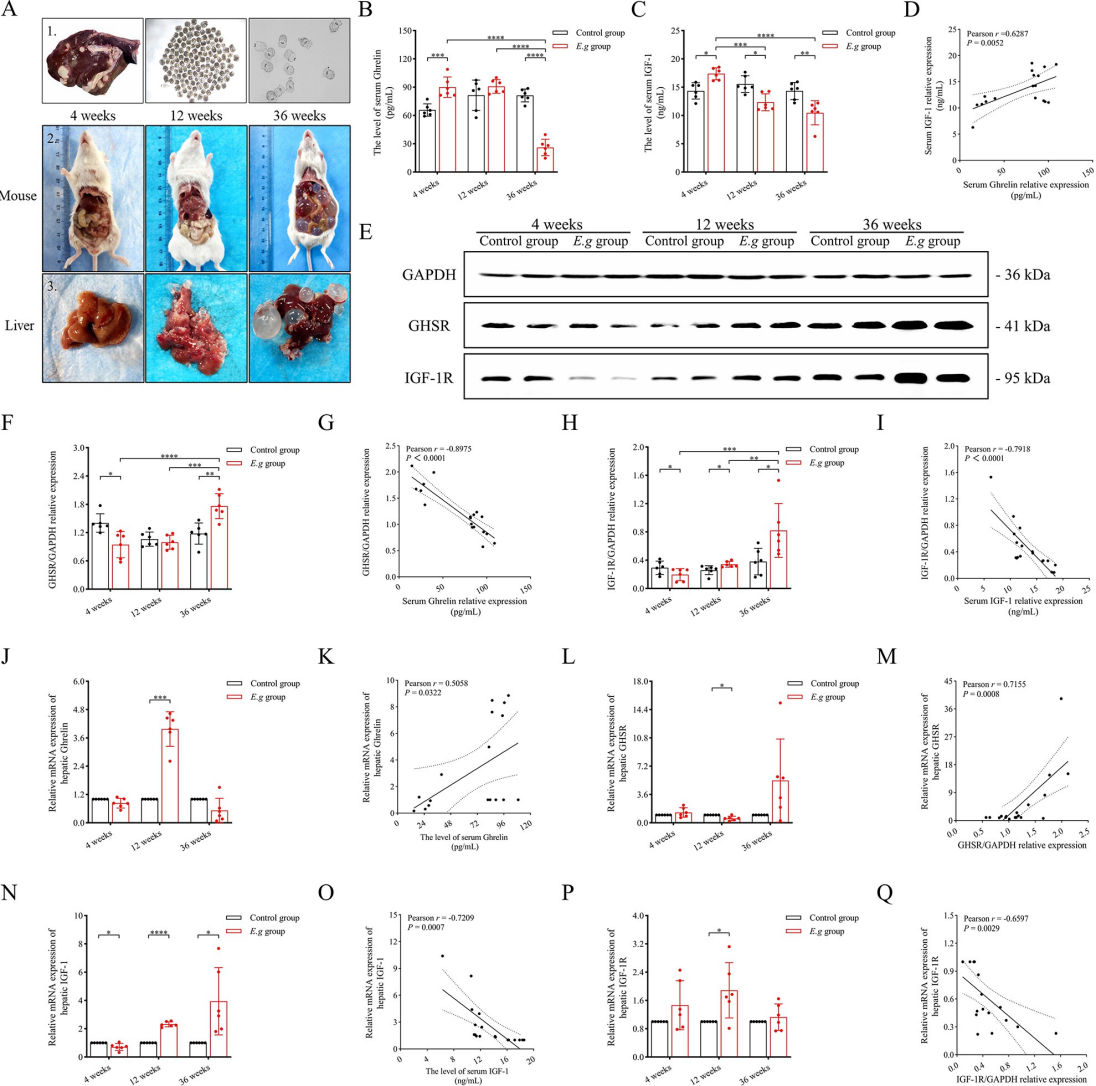
The expression of Ghrelin, IGF-1 and their receptors in the serum and liver of *E*.*g*-infected mice at different stages of infection. (A) showed *E*.*g*-infected sheep livers and PSCs with >95% activity isolated from liver cystic lesions (A1), and showed abdominal anatomy (A2) and liver lesion growth (A3) of *E*.*g*-infected mice at 4, 12, and 36 weeks. Serum levels of Ghrelin (B) and IGF-1 (C) were analyzed by ELISA in mice, and correlation analysis was performed (D). (E) showed images of the WB strip of mouse liver, and the protein levels of GHSR (F) and IGF-1R (H) were analyzed using GAPDH as an internal reference, and correlation analyses were performed between the serum levels of Ghrelin and relative protein levels of GHSR (G), and the serum levels of IGF-1 and relative protein levels of IGF-1R (I). In addition, RT-qPCR was performed to analyze the relative mRNA levels of Ghrelin (J), GHSR (L), IGF-1 (N), IGF-1R (P), and to perform correlation analysis between the Ghrelin serum level and relative mRNA level (K), the GHSR relative protein level and relative mRNA level (M), the IGF-1 serum level and relative mRNA level (O), the IGF-1R relative protein level and relative mRNA levels (Q). Data was considered statistically significant: * *P* < 0.05; ** *P* < 0.01; *** *P* < 0.001; **** *P* < 0.0001. The author is the photographer of the image, and the original image has no copyright dispute.

Fresh livers of *E*.*g*-infected sheep were obtained from livestock slaughterhouses around Urumqi, Xinjiang Uygur Autonomous Region, China, and PSCs were collected from cystic lesions on the surface of the livers and added to RPMI-1640 complete medium solution [70 mL of RPMI-1640 medium solution (Thermo Fisher Scientific, MA, USA) + 20 mL of 10% Fetal Bovine Serum (FBS) (Cytiva, Shanghai, China) + 8 mL of 5% yeast extract solution + 2 mL of 0.9% sterile saline containing 2% 100 IU/mL penicillin and 100 IU/mL streptomycin solution], and incubated in a 37°C 5% CO_2_ thermostat. When used, an appropriate amount of PSCs was taken and stained with 0.1% eosin staining to observe the activity under an inverted microscope. The active PSCs were not stained, and the immature and dead PSCs were red. The total number and proportion of unstained and stained PSCs were calculated and PSCs with activity >95% were used for animal modelling and *in vitro* drug intervention experiments, (Fig 1A.1).

### Preparation and experimental design of the hepatic *E*.*g*-infected model

Surgery for the preparation of the BALB/c mouse model of hepatic *E*.*g* infection was performed using standard aseptic techniques [60]. Mice were anesthetized by ether inhalation. A small incision of 1 cm was made longitudinally in the middle of the upper abdomen into the abdominal cavity, and the intestinal canal was dissected to reveal the portal vein. 100 μL of 0.9% sterile saline containing 2000 PSCs was injected into the portal vein with a scalp needle. The abdominal incision was closed with a 6~0 absorbable suture after 3 min of light pressure to stop bleeding. Diet was resumed 12 hours after surgery.

BALB/c mice were randomly divided into 6 groups according to whether they were infected with *E*.*g* and the length of rearing: Control group and *E*.*g* group (6 mice per group) at 4 weeks, 12 weeks and 36 weeks. Mice were euthanized by ether inhalation anesthesia and cervical dislocation, and serum and liver tissue samples were collected.

### Drug intervention experiment with HepG2 cells and PSCs protein co-cultured in vitro

PSCs and enzyme-free sterile PBS solution were mixed 1:1, homogenized in a cryomill and shaken overnight at 4°C. The supernatant containing PSCs protein was collected by centrifugation in a low-temperature, high-speed centrifuge (10,000 rpm, 4°C, 10 min) on the following day. The *in vitro* medium solution was DMEM basic solution (Thermo Fisher Scientific, MA, USA) supplemented with 10% FBS (Cytiva, Shanghai, China), 0.9% sterile saline solution containing 2% 100 IU/mL penicillin and 100 IU/mL streptomycin, and *in vitro* culture was performed at 37°C in a 5% CO_2_ thermostat. Firstly, laboratory passaged preserved HepG2 cells and different concentrations (40 μg/mL, 60 μg/mL, 80 μg/mL, and 100 μg/mL) of PSCs protein were co-cultured *in vitro* for 24 h. The HepG2 group was set up, and the supernatant and the lower cell extracts were collected. Next, HepG2 cells and PSCs protein (100 μg/mL) were co-cultured *in vitro* for 24 h using Ghrelin protein [Ghrelin protein(Uniprot No. Q30DT1) was synthesized manually by Fmoc (9-fluorenylmethoxycarbonyl) solid-phase peptide synthesis procedures, and purified by reverse-phase HPLC (TSK gel ODS-120A column; linear gradient of 0%-60% CH3CN in the presence of 0.1% trifluoroacetic acid), as a gift from Professor Hideto Kuwayama, at Obihiro University of Agriculture and Veterinary Medicine, Japan.] and [D-Lys3]-GHRP-6 (Abcam, Cambridge, MA, USA) for intervention. Ghr^+^ was used to denote Ghrelin protein and Ghr^-^ to denote [D-Lys3]-GHRP-6. According to the concentration and duration of intervention, they were divided into HepG2 group, HepG2+*E*.*g* group, HepG2+*E*.*g*+Ghr^+^ (400 ng/mL, 8 h) group, HepG2+*E*.*g*+Ghr^+^ (600 ng/mL, 8 h) group, HepG2+*E*.*g*+Ghr^+^ (400 ng/mL, 16 h) group, HepG2+*E*.*g*+Ghr^+^ (600 ng/mL, 16 h) group, HepG2+*E*.*g*+Ghr^-^ (400 ng/mL, 8 h) group, HepG2+*E*.*g*+Ghr^-^ (600 ng/mL, 8 h) group, and HepG2+*E*.*g*+ Ghr^-^ (400 ng/mL, 16 h) group, HepG2+*E*.*g*+Ghr^-^ (600 ng/mL, 16 h) group, and supernatant and lower cell extracts were collected.

### Drug intervention experiment in PSCs in vitro culture

*In vitro* culture of PSCs was carried out in 96-well culture plates. Two hundred PSCs were added into each well and divided into six groups according to the different intervention methods: Control group (RPMI-1640 complete medium 200 μL), Ghrelin group (RPMI-1640 complete medium 198 μL + Ghrelin protein 2 μL), [D-Lys3]-GHRP-6 group (RPMI-1640 complete medium 198 μL + [D-Lys3]-GHRP-6 2 μL), Albendazole sulfoxide (ABZSX) group (RPMI-1640 complete medium 198 μL + ABZSX 2 μL), [D-Lys3]-GHRP-6 + ABZSX group (RPMI-1640 complete medium 198 μL + [D-Lys3]-GHRP-6 1 μL + ABZSX 1 μL). The drug concentration of Ghrelin protein and [D-Lys3]-GHRP-6 was 100 μmol/L, and that of ABZSX (kept in our laboratory) was 10 μmol/L. The survival rate of PSCs was observed under an inverted microscope by 0.1% eosin staining, and the microstructure of PSCs was observed under a scanning electron microscopy (JEOL, Japan).

### Enzyme-linked immunosorbent assay (ELISAs) and liver enzyme assays

ELISA was used to quantify the levels of Ghrelin and IGF-1 in mice serum, HepG2 cell culture supernatant and cell extract homogenate, the levels of monoamine oxidase (MAO), prolyl hydroxylase (PH), TGF-β1, IL-2, INF-γ, TNF-α, IL-4, IL-6 and IL-10 in HepG2 cell culture supernatant. ELISA methods were all performed according to the operating instructions and determined by enzyme labeling instrument (BioTek, Vermont, USA). All ELISA kits were purchased from Lapuda Biotechnology (Nanjing, China) except for PH which was purchased from Jin Yibai Biotechnology (Nanjing, China).

### Western blotting (WB)

Liver tissues or cells were homogenized by adding RIPA lysate in a cryomill. The homogenized solution was further vortexed with phosphatase inhibitor solution diluted at 1:100 and normal-type protease inhibitor solution diluted at 1:100 and allowed to stand for 40 min, then centrifuged at low temperature and high speed (12000 rpm, 4°C, 20 min), and the protein supernatant was collected. Mice liver tissue proteins were sampled at a total of 60 μg/well and cell extract proteins were sampled at a total of 30 μg/well. Proteins were separated using 10% SDS-PAGE gels (Biotides, Beijing, China) and membranes were electrotransfected with PVDF electrotransfer membranes (Sigma-Aldrich, Shanghai, China). The membranes were treated with 5% skimmed milk powder sealing solution and then incubated with primary antibody solution at 4°C overnight. Goat anti-rabbit HRP-IgG antibody was used for secondary antibody incubation. Then, the gel imaging system (Bio-Rad, Hercules, USA) was used for color development analysis after immersion with ECL chemiluminescent agent solution (Beyotime Biotechnology, Shanghai, China) protected from light. The WB bands were quantified using Image Lab 5.1 software (BIO-RAD, Shanghai, China). Among the reagents used, RIPA lysate was purchased from Solarbio (Beijing, China), phosphatase inhibitors, general protease inhibitors, Goat anti-rabbit HRP-IgG antibody, Anti-β-actin, Anti-GAPDH, Anti-Myd88, Anti-TLR4, Anti-cGAS and Anti-Sting were purchased from Proteintech (Wuhan, China), Anti-GHSR and Anti-Smad3 were purchased from Affinity Biosciences (OH, USA), Anti-IGF-1R was purchased from Cell Signaling (MA, USA), NF-κB p65, Anti-Cyclin D1 and Anti-Cyclin E1 were purchased from Abcam (Cambridge, MA, USA).

### Reverse transcription quantitative real-time polymerase chain reaction (RT-qPCR)

Total RNA was extracted from mouse liver tissue homogenates by adding Triquick Reagent (Solarbio, Beijing, China) and reverse transcribed using the UnionScript First-strand cDNA Synthesis Mix for qPCR kit (Genesand Biotech, Beijing, China). The GS AntiQ qPCR SYBR Green Fast Mix kit (Genesand Biotech, Beijing, China) and the ABI Prism 7500 Real-Time Fluorescent Quantitative PCR System (Applied Biosystems, CA, USA) were used for the detection (Holding Stage: 94°C for 30 s, Cycling Stage: 94°C for 5 s for 40 cycles, 60°C for 34 s for 40 cycles, Melting Curve Stage: 95°C for 15 s, 60°C for 60 s, 95°C for 30 s and 60°C for 15 s). The relative mRNA expression of Ghrelin (5′-GCACCAGAAAGCCCAGAGAAAGG-3′ forward and 5′-TCTCTTCTGCTTGTCCTCTGTCCTC-3′ reverse), GHSR (5′-GAGCACGAGAACGGCACAGATC-3′ forward and 5′-ACACCACCACAGCAAGCATCTTC-3′ reverse), IGF-1 (5′-GCTCTGCTTGCTCACCTTCACC-3′ forward and 5′-AACACTCATCCACAATGCCTGTCTG-3′ reverse) and IGF-1R (5′-GCCAACAAGTTCGTCCACAGAG-3′ forward and 5′-GGTAGTAGTCCGTCTCGTAGATGTC-3′ reverse) was analyzed by the 2^-△△Ct^ method using GAPDH as an internal reference gene.

### Polymerase chain reaction (PCR)

PCR amplification of PSCs was performed using Ghrelin primers (5′-TACTACTCTCCACGCCC-3′ forward and 5′-AGGGGCCATCCACAGTCTTC-3′ reverse). The amplified products were subjected to agarose gel electrophoresis (conditions: voltage 120 V, current 100 mA, 30 min). The gene expression of Ghrelin was detected by electrophoretic analysis using a gel imaging system (Tanon, Shanghai, China).

### Immunohistochemistry (IHC)

After paraffin slices of liver tissue were deparaffinized and treated with gradient dehydration, the detection of Ghrelin and GHSR was treated with citrate buffer (pH 6.0), and IGF-1 and IGF-1R were treated with Tris-EDTA buffer (Thermo Fisher Scientific, MA, USA) for microwave antigen repair for 15 min. The paraffin slices were incubated dropwise with the primary antibody solution at 4°C overnight after closed by the sheep serum working solution (ZSGB-Bio, Beijing, China). Goat anti-rabbit/mouse HRP-labelled polymer (Proteintech, Wuhan, China) was used for secondary antibody incubation, the DAB kit for color development and hematoxylin solution (ZSGB-Bio, Beijing, China) for staining. Observation was performed using a light microscope (Cat #BX43, Olympus, Japan). All pathological slices were randomly captured under a light microscope for images of 3 fields of lesion view, and the integral optical density (IOD) and area of IHC protein expression were quantified using Image-Pro Plus 6.0 imaging software, and the average optical density (AOD) was calculated by virtue of them. The reagents used in IHC include Anti-Ghrelin and Anti-NF-κB p65 purchased from Abcam (Cambridge, MA, USA), Anti-IGF-1, Anti-IGF-1R, Anti-Myd88, HRP-labeled polymer, and DAB kit were purchased from Proteintech (Wuhan, China), and Anti-GHSR purchased from Affinity Biosciences (OH, USA).

### Fluorescence-based multiplex immunohistochemistry (mIHC) staining

After deparaffinised and treated with gradient dehydration, the paraffin slices of mice liver tissue were microwaved in citrate buffer (pH 6. 0) for 8 min on medium heat, cease fire for 8 min, and transferred to medium-low heat for 7 min for antigen repair. The paraffin slices were incubated dropwise with Anti-Ghrelin at 4°C overnight after being closed by the serum working solution. The corresponding HRP-labeled secondary antibody solution was incubated for 50 min and the tyramide signal amplification fluorescent dye was incubated for 10 min away from light, sequentially. After microwave repair, the above steps were repeated to complete the incubation of Anti-GHSR, HSCs marker Anti-α-SMA, T-cell marker Anti-CD3, macrophage marker Anti-CD68), hepatocyte marker Anti-albumin, Anti-Myd88, Anti-NF-κB p65, Anti-TGF-β1 and Anti-Smad3. Finally, they were incubated with DAPI for 10 min in the dark to stain the nucleus and anti-fluorescence quencher seal. The expression was observed under a fluorescence microscope (Nikon, Japan) and scanner (3DHISTECH, Hungary). The Pearson’s correlation coefficient (r) and overlap coefficient were analyzed for mIHC images using Image-Pro Plus 6.0 imaging software. Among the reagents used in mIHC, except Anti-ghrelin purchased from Abcam (Cambridge, MA, USA) and Anti-GHSR purchased from Affinity Biosciences (OH, USA), all others were purchased from Servicebio (Wuhan, China).

### Immune electron microscopy (IEM)

Fresh liver tissue samples from mice were cut into 3 mm^3^ pieces and fixed in EP tubes containing immunoelectron microscopy fixative (Servicebio, Wuhan, China) at 4°C for 48 h. Then, fresh liver tissue samples were resin infiltrated and embedded at 4°C after being rinsed with pre-cooled 0.1 M phosphate buffer (pH 7.4) at 4°C and treated with gradient dehydration. Samples (70~80 nm) were cut on an ultrathin microtome (Leica, Germany) and slices were fished with nickel mesh (EMCN, Beijing, China) for immunolabelling. The slices were blocked with 1% BSA/TBS blocking solution for 30 min at room temperature, and incubated with Anti-Ghrelin (Abcam, Cambridge, MA, USA) diluted 1:50 with the blocking solution at 4°C overnight. The following day, the slices were incubated with secondary antibody [12 nm colloidal gold Goat Anti-Rab (Jackson, CA, USA)] diluted 1:25 with diluent solution at room temperature for 20 min, then incubated in the oven at 37°C for 60 min, and then rewarmed at room temperature for 30 min. The nickel mesh with the sample was stained with 2% saturated alcoholic solution of uranyl acetate for 15 min in a light-proof manner, and then placed in the oven at 37°C for 10 min. After that, the images were acquired using a transmission electron microscope (Hitachi, Japan), and the black, 12 nm-sized particles were considered to have a positive expression.

### Cell counting kit-8 (CCK-8)

The activity test for drug intervention in HepG2 cell culture *in vitro* was performed according to the instruction of CCK-8 kit (Yeasen Biotechnology, Shanghai). The plates were incubated at 37°C in a 5% CO_2_ incubator for 3 h, and then the absorbance at 450 nm was measured by an enzyme marker.

### Statistical analysis

All data were statistically analyzed using Graphpad Prism 9.5 software. Measured data were expressed as means ± standard deviation (SD) and count data were expressed as rate or constitutive ratio. The *t*-test was used to compare measures between two groups, the Chi-square test was used to compare count data, and the One-way ANOVA was used to compare measures between multiple groups. Pearson correlation coefficients (r) and simple linear regression were used to analyze the associations between variables. Data was considered statistically significant: * *P* < 0.05; ** *P* < 0.01; *** *P* < 0.001; **** *P* < 0.0001.

## Results

### Serum Ghrelin levels in *E*.*g*-infected mice were elevated early in the disease, decreased later, and negatively regulated receptor proteins in the liver

In this study, the liver lesions progressed as seen by the naked eye as the duration of infection in *E*.*g*-infected mice increased, (Fig 1A.2, 3). ELISA showed that the serum Ghrelin levels in the *E*.*g* group were significantly elevated at 4 weeks (*P* = 0.0008), elevated but not significant at 12 weeks and significantly decreased at 36 weeks (*P* < 0.0001) compared to the control group during the same period. The intergroup comparisons of the *E*.*g* group showed a significantly lower level at 36 weeks than at 4 weeks and 12 weeks (*P* < 0.0001), (Fig 1B). Serum IGF-1 levels in the *E*.*g* group were significantly higher at 4 weeks (*P* = 0.0103) and significantly lower at 12 weeks and 36 weeks (*P* = 0.0104, *P* = 0.0034, respectively) compared to the control group over the same period, and the intergroup comparisons of the *E*.*g* group showed significantly lower levels at 12 weeks and 36 weeks than at 4 weeks (*P* < 0.0001) (Fig 1C). The correlation analysis of serum Ghrelin and IGF-1 levels in the *E*.*g* group showed a significant positive regulatory relationship between the two (*r* = 0.6287, *P* = 0.0052), (Fig 1D). Our previous studies defined 180 days as the cut-off point between the early and late stages of *E*.*g* infection based on trends in metabolic, apoptotic and fibrotic factors [61,62]. These data showed that the serum Ghrelin and IGF-1 levels were elevated in the early stage of infection and decreased in the late stage of infection in *E*.*g*-infected mice. WB showed that the relative protein levels of hepatic GHSR in the *E*.*g* group was significantly lower at 4 weeks (*P* = 0.0405) and higher at 36 weeks compared to the control group over the same period (*P* = 0.0031). The intergroup comparisons of the *E*.*g* group showed a significantly higher level at 36 weeks than at 4 weeks and 12 weeks (*P* < 0.0001). The relative protein levels of hepatic IGF-1R were significantly lower at 4 weeks (*P* = 0.0169) and higher at 12 weeks and 36 weeks (*P* = 0.015, *P* = 0.0165, respectively) compared to the control group over the same period of time, and the intergroup comparisons of the *E*.*g* group showed 36 weeks to be significantly higher than 4 weeks and 12 weeks (*P* = 0.0031). higher than 4 weeks and 12 weeks (*P* = 0.0007). The results of correlation analysis showed a significant negative regulatory relationship between changes in serum Ghrelin and IGF-1 levels and hepatic protein levels of their respective receptors in the *E*.*g* group (*r* = -0.8975, *P* < 0.0001 and *r* = -0.7918, *P* < 0.0001, respectively), (Fig 1E–1I). In addition, we examined the mRNA relative expression of hepatic Ghrelin, IGF-1 and their receptors by RT-qPCR. The results showed a significant positive regulatory relationship between Ghrelin, GHSR protein expression levels and mRNA relative expression in *E*.*g* group (*r* = 0.5058, *P* = 0.0322 and *r* = 0.7155, *P* = 0.0008, respectively), and a significant negative regulatory relationship between IGF-1, IGF-1R protein expression levels and mRNA relative expression (r = -0.7209, *P* = 0.0007 and r = -0.6597, *P* = 0.0029, respectively), (Fig 1J–1Q).

### *E*.*g*-infected mice consistently recruited ghrelin and its receptor in and around liver lesions

**Fig 2:**
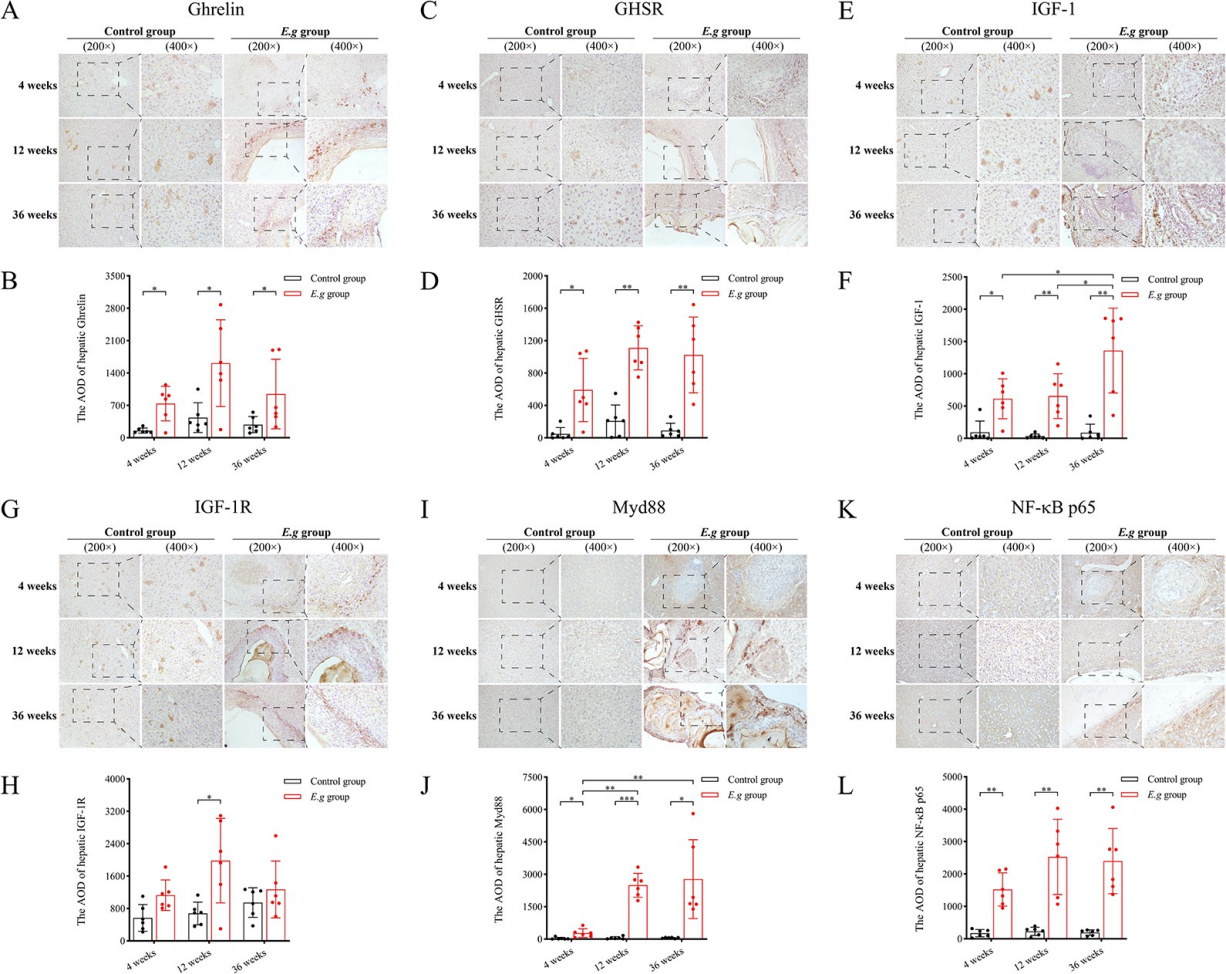
The protein expression of Ghrelin, IGF-1, their receptors, Myd88 and NF-κB p65 in and around liver lesions at different stages of infection in *E*.*g*-infected mice. IHC analyses were performed on liver slices from the 4 weeks, 12 weeks, 36 weeks control groups and *E*.*g* groups. Where (A) showed IHC stained images of Ghrelin, (C) showed IHC stained images of GHSR, (E) showed IHC stained images of IGF-1, (G) showed IHC stained images of IGF-1R, (I) showed IHC stained images of Myd88, and (K) showed NF-κB p65 IHC staining images. The AOD of IHC staining images of Ghrelin (B), GHSR (D), IGF-1 (F), IGF-1R (H), Myd88 (J), and NF-κB p65 (L) were quantified and analyzed using Image-Pro Plus 6.0 imaging software. Data was considered statistically significant: * *P* < 0.05; ** *P* < 0.01; *** *P* < 0.001; **** *P* < 0.0001. AOD: average optical density.

We observed by IHC that the protein expression of Ghrelin, GHSR, and IGF-1 was significantly elevated in and around the liver lesions of the *E*.*g* group compared with that of the control group at the same period of time at 4 weeks, 12 weeks, and 36 weeks (Ghrelin: *P* = 0.0138, *P* = 0.0106, *P* = 0.0394, respectively; GHSR: *P* = 0.0212, *P* = 0.0014, *P* = 0.0064, respectively and IGF-1: *P* = 0.0223, *P* = 0.0055, *P* = 0.0073, respectively), (Fig 2A–2F). The protein expression of IGF-1R was significantly elevated only at 12 weeks (*P* = 0.0313), (Fig 2G and 2H). In addition, the intergroup comparisons of *E*.*g* groups showed that the protein expression of Ghrelin in and around the liver lesions started to show a decreasing trend at 36 weeks, but there was no significant difference, (Fig 2A and 2B). The persistently high protein expression of GHSR and IGF-1 didn’t show a decreasing trend, (Fig 2C–2F). In addition, the protein expression of Myd88 and NF-κB p65 was also significantly elevated in and around the liver lesions of the *E*.*g* group compared to the control group during the same period at 4 weeks, 12 weeks, and 36 weeks (Myd88: *P* = 0.0471, *P* = 0.0001, *P* = 0.0153, respectively; NF-κB p65: *P* = 0.0016, *P* = 0.0039, *P* = 0.0027, respectively), and the intergroup comparisons of the *E*.*g* group showed that the protein expression of Myd88 was significantly higher at 12 weeks and 36 weeks than at 4 weeks (*P* = 0.0025), (Fig 2I–2L)

### Highly expressed Ghrelin in and around liver lesions of *E*.*g*-infected mice was involved in the regulation of TGF-β1/Smad3 and Myd88/NF-κB signal pathway

**Fig 3:**
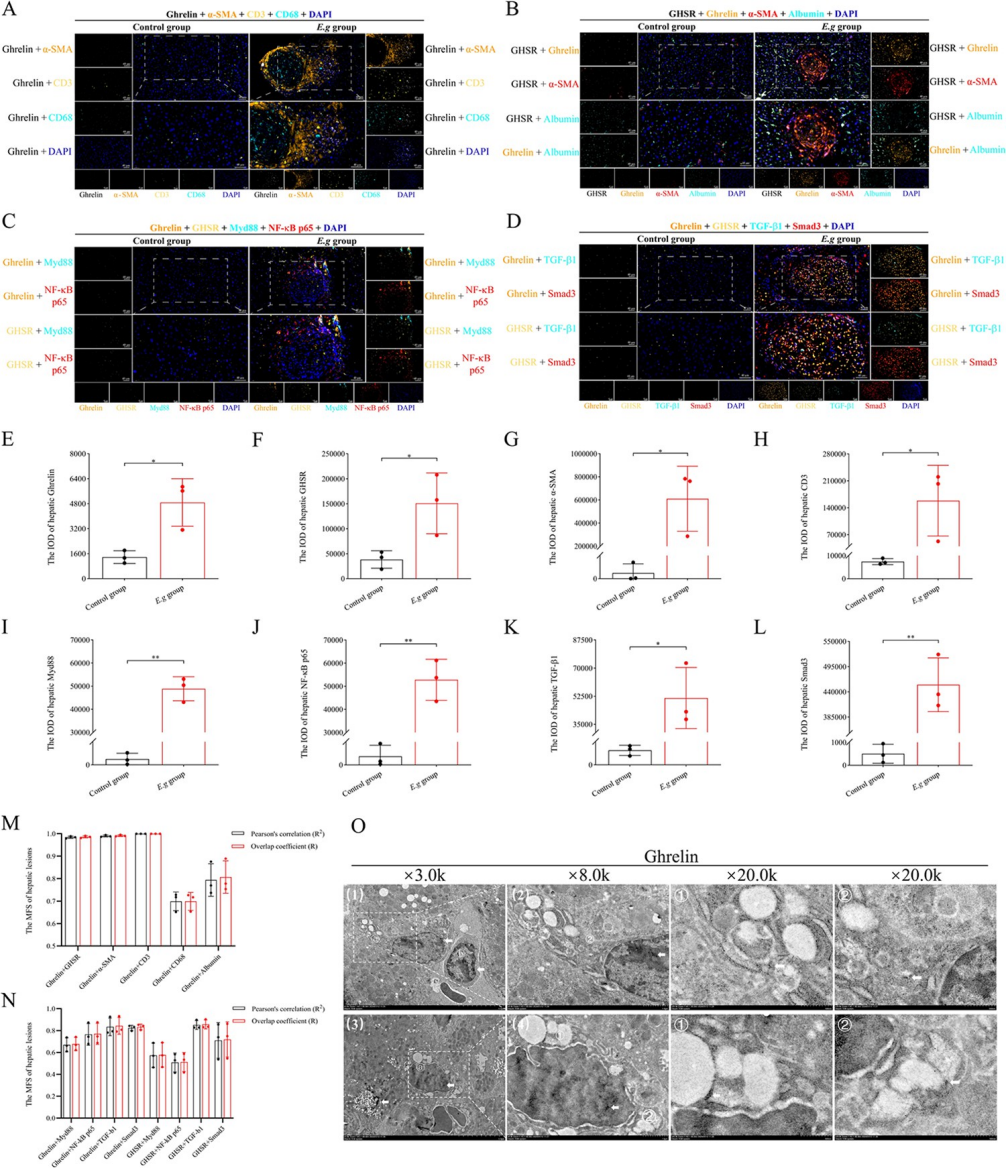
Correlation analysis between the protein expression of Ghrelin and immunoinflammation, fibrotic cytokines in and around liver lesions of *E*.*g*-infected mice. mIHC analysis of liver slices from the 12-week control group and *E*.*g* group was performed. (A) showed the mIHC-stained images of Ghrelin and α-SMA, CD3, CD68, DAPI. (B) Showed the mIHC-stained images of Ghrelin and GHSR, α-SMA, Albumin, DAPI. (C) Showed the mIHC-stained images of Ghrelin and GHSR, Myd88, NF-κB, DAPI. (D) Showed the mIHC-stained images of Ghrelin and GHSR, TGF-β1, Smad3, DAPI. Quantification analysis of the IOD of mIHC-stained images of Ghrelin (E), GHSR (F), α-SMA (G), CD3 (H), Myd88 (I), NF-κB (J), TGF-β1 (K), Smad3 (L) in and around liver lesions of *E*.*g*-infected mice was performed and the Pearson correlation coefficients and overlap coefficients (M, N) of their protein expression were analyzed by Image-Pro Plus 6.0 imaging software. (O) showed IEM observations of the protein expression of Ghrelin present on lysosomes (1ⓐ, 3ⓐ) and inflammatory nuclei (1ⓑ), and black dots indicated by white arrowheads are Ghrelin-positive expression. Data was considered statistically significant: * *P* < 0.05; ** *P* < 0.01; *** *P* < 0.001; **** *P* < 0.0001. IOD: integrated optical density.

The previous studies had reported the protein expression of Ghrelin in hepatocytes and HSCs [63]. In this study, we found significantly higher protein expression of Ghrelin, GHSR, α-SMA and CD3 in and around the liver lesions of the *E*.*g* group at 12 weeks compared to the control group at the same time by mIHC (*P* = 0.0185, *P* = 0.0371, *P* = 0.0198, *P* = 0.0465, respectively), (Fig 3A, 3B, 3E–3H). Co-localization analysis indicated that the protein expression of Ghrelin showed an extremely strong co-localization with GHSR, a-SMA, CD3 (GHSR: Pearson’s correlation R^2^ = 0.9842±0.0050, Overlap coefficient R = 0.9858±0.0047; a-SMA: Pearson’ s correlation R^2^ = 0.9898±0.0041, Overlap coefficient R = 0.9915±0.0038; CD3: Pearson’s correlation R^2^ = 1.0000±0.0000, Overlap coefficient R = 1.0000±0.0000), and a strong co-localization with CD68 and Albumin (CD68: Pearson’s correlation R^2^ = 0.6990±0.0417, Overlap coefficient R = 0.6988±0.0399; Albumin: Pearson’s correlation R^2^ = 0.7941±0.0725, Overlap coefficient R = 0.8069±0.0722), (Fig 3M). In addition, the protein expression of Myd88, NF-κB p65, TGF-β1 and Smad3 in the liver lesions and periphery of the *E*.*g* group was also significantly elevated compared with that of the control group during the same period (*P* = 0.0039, *P* = 0.0098, *P* = 0.0466, *P* = 0.0056, respectively), (Fig 3C, 3D, 3I–3L). Co-localization analysis indicated that the protein expression of Ghrelin showed an extremely strong co-localization with TGF-β1, Smad3 (TGF-β1: Pearson’s correlation R^2^ = 0.0.8343±0.0802, Overlap coefficient R = 0.8438±0.0767; Smad3: Pearson’s correlation R^2^ = 0.8245±0.0240, Overlap coefficient R = 0.8339±0.0254), and a strong co-localization with Myd88 and NF-κB p65 (Myd88: Pearson’s correlation R^2^ = 0.6698±0.0640, Overlap coefficient R = 0.6776±0.0605; NF-κB p65: Pearson’s correlation R^2^ = 0.7656±0.0992, Overlap coefficient R = 0.7707 ± 0.0964). The protein expression of GHSR showed an extremely strong co-localization with TGF-β1 (Pearson’s correlation R^2^ = 0.8517 ± 0.0405, Overlap coefficient R = 0.8568 ± 0.0406), a strong co-localization with Smad3 (Pearson’s correlation R^2^ = 0.7104 ± 0.1618, Overlap coefficient R = 0.7191 ± 0.1604), and a moderately strong co-localization with Myd88 and NF- κB p65 (Myd88: Pearson’s correlation R^2^ = 0.5739 ± 0.1120, Overlap coefficient R = 0.5774 ± 0.1120; NF-κB p65: Pearson’s correlation R^2^ = 0.5087±0.0865, Overlap coefficient R = 0.5125±0.0869), (Fig 3N). In addition, we also observed the presence of protein expression of Ghrelin in inflammatory cell nuclei and lysosomes by IEM, (Fig 3O).

### PSCs protein of *E*.*g* could stimulate Ghrelin secretion from HepG2 cells in a concentration-dependent manner in *in vitro* experiment

**Fig 4:**
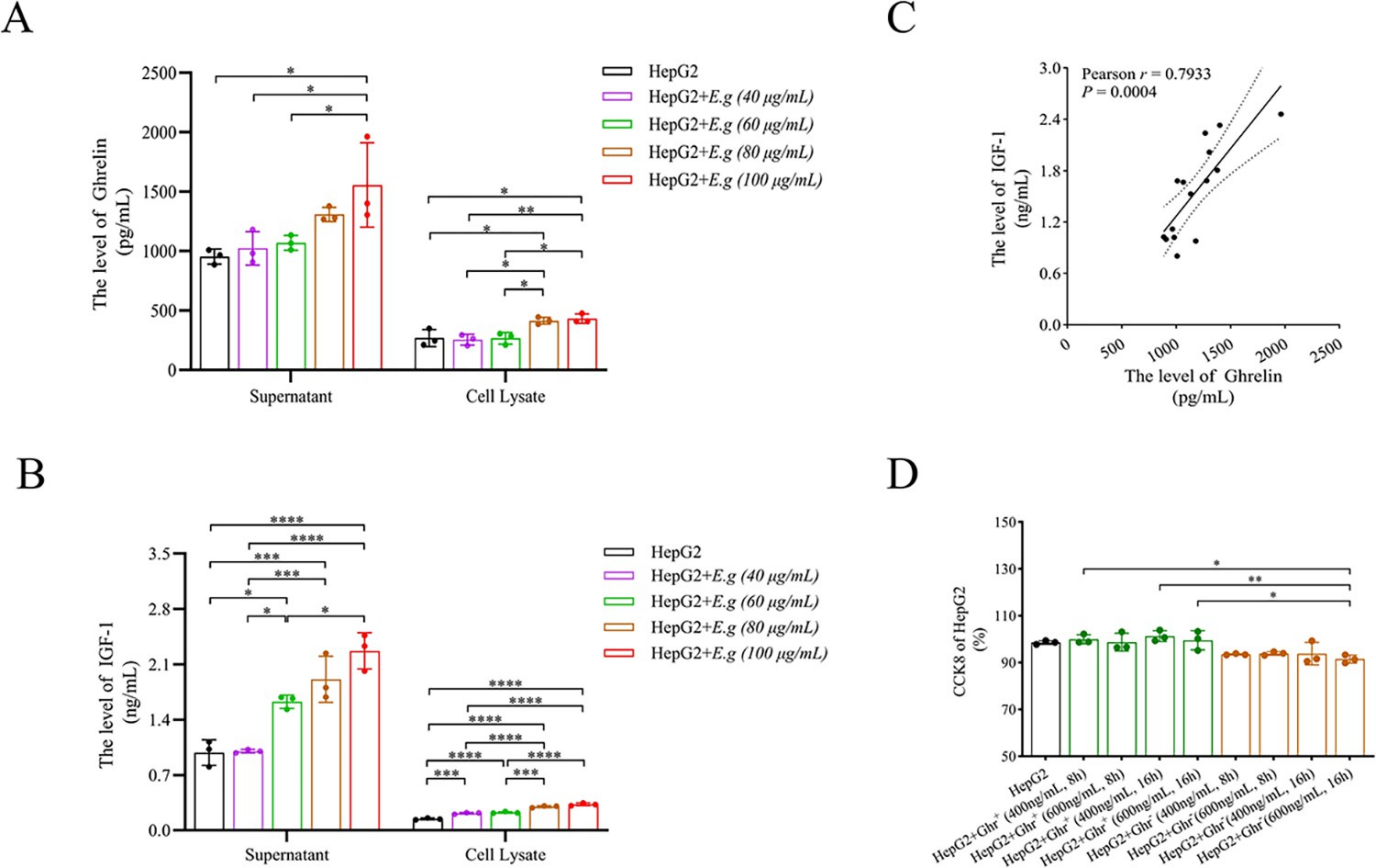
The expression of Ghrelin in HepG2 cells and PSCs protein co-cultured *in vitro*. The levels of Ghrelin (A) and IGF-1 (B) in the supernatant and cell extracts of HepG2 cells co-cultured with PSCs protein of different concentrations were analyzed by ELISA, and a correlation analysis of the supernatant levels was performed (C). (D) showed the effects of different intervention concentrations and times of Ghrelin and [D-Lys3]-GHRP-6 on the viability of HepG2 cells using CCK-8. Data was considered statistically significant: * *P* < 0.05; ** *P* < 0.01; *** *P* < 0.001; **** *P* < 0.0001.

First, we co-cultured HepG2 cells with different concentrations of PSCs proteins, and ELISA showed that PSCs protein stimulation of HepG2 cells was able to increase the secretion of both Ghrelin and IGF-1, and the secretion increased with the rising concentration, (Fig 4A and 4B). Correlation analysis of the supernatant ELISA also showed a significant positive correlation between the two (*r* = 0.7933, *P* = 0.0004), (Fig 4C). We also found that PSCs protein stimulation at 100 μg/mL could simultaneously cause significant increases in the supernatant levels of Ghrelin and IGF-1 (*P* = 0.0108, *P* < 0.0001, respectively), and that PSCs protein stimulation at 80 μg/mL could simultaneously cause significant increases in the lower cellular extract levels of Ghrelin and IGF-1 (*P* = 0.0018, *P* < 0.0001, respectively), (Fig 4A and 4B). In addition, we examined the effects of different intervention concentrations and times of Ghrelin and [D-Lys3]-GHRP-6 on the viability of HepG2 cells using CCK-8. The results showed that 400 ng/mL and 600 ng/mL of Ghrelin protein and [D-Lys3]-GHRP-6 intervened for 8 h and 16 h didn’t significantly affect the viability of HepG2 cells, (Fig 4D).

### High expression of Ghrelin could ameliorate immunoinflammation and fibrosis induced by PSCs protein stimulation in *in vitro* cultures of HepG2 cells

**Fig 5:**
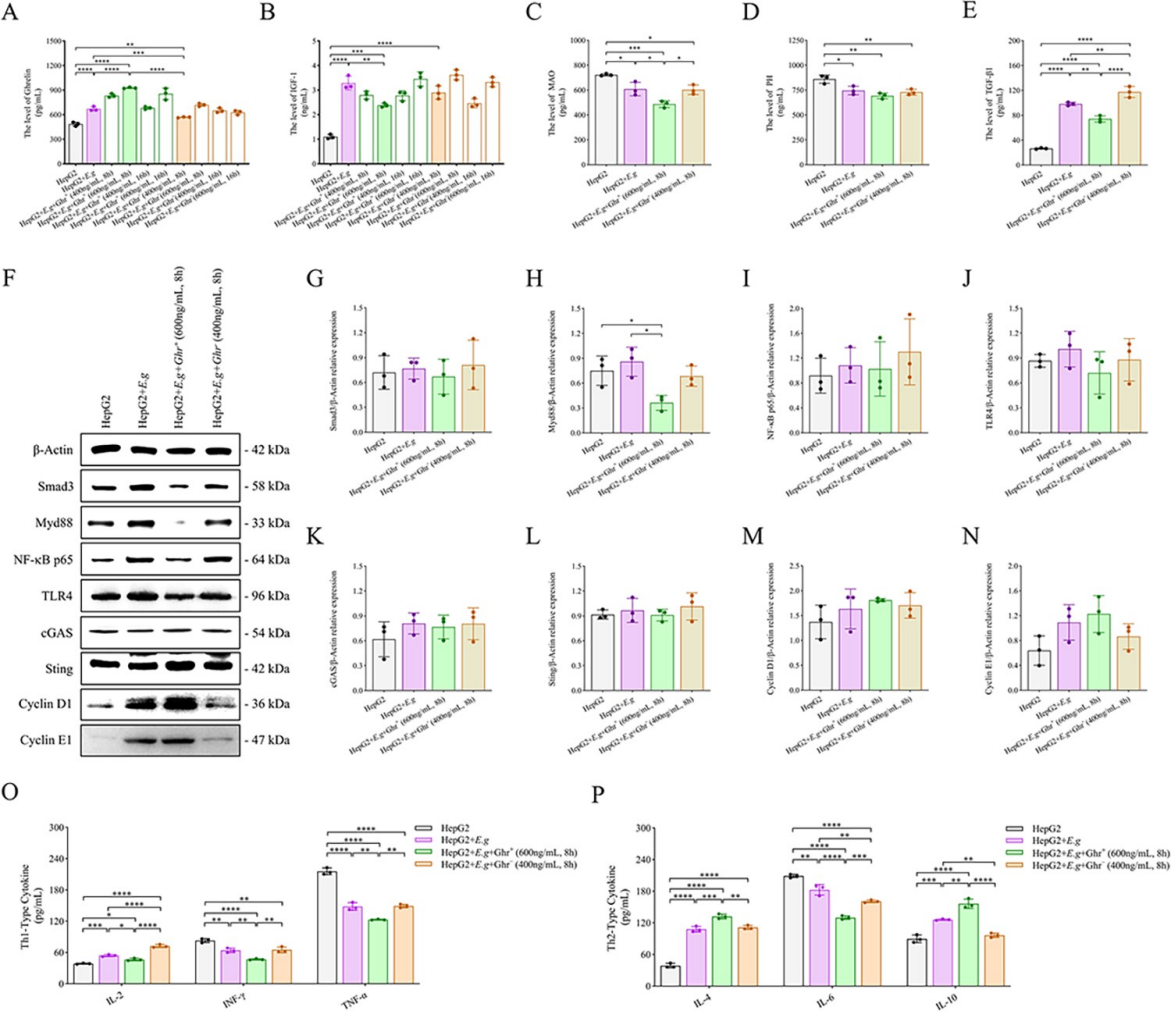
The expression of Ghrelin and immunoinflammatory, fibrotic cytokines in HepG2 cells and PSCs protein co-cultured *in vitro*. HepG2 cells and 100 μg/mL of PSCs protein co-culture medium was intervened using different concentrations of Ghrelin protein and [D-Lys3]-GHRP-6 for different durations, and the levels of supernatant Ghrelin (A) and IGF-1 (B) were analyzed by Elisa. HepG2 group, HepG2+*E*.*g* group, HepG2+*E*.*g*+Ghr^+^ (600 ng/mL, 8 h) group and HepG2+*E*.*g*+Ghr^-^ (400 ng/mL, 8 h) group were selected for comparative analyses, and supernatants were analyzed by ELISA for the levels of MAO (C), PH (D), TGF-β1 (E), Th1-type cytokines (IL-2, IFN-γ and TNF-α) (O) and Th2-type cytokines (IL-4, IL-6 and IL-10) (P). (F) showed the WB strip images of cell extracts and the relative protein levels of Smad3 (G), Myd88 (H), NF-κB p65 (I), TLR4 (J), cGAS (K), Sting (L), Cyclin D1 (M) and Cyclin E1 (N) were analyzed using β-Actin as an internal reference. Data was considered statistically significant: * *P* < 0.05; ** *P* < 0.01; *** *P* < 0.001; **** *P* < 0.0001. PSCs: protoscoleces. MAO: monoamine oxidase. PH: prolyl hydroxylase.

We further intervened with HepG2 cells and 100 μg/mL of PSCs protein co-culture medium using different concentrations and times of Ghrelin protein and [D-Lys3]-GHRP-6, and comparing to the HepG2 group and the HepG2+*E*.*g* group. The supernatant ELISA showed that the Ghrelin levels in the HepG2+*E*.*g*+Ghr^+^ (600 ng/mL, 8 h) group and HepG2+*E*.*g*+Ghr^-^ (400 ng/mL, 8 h) group had the most significant changes (*P* < 0.0001), (Fig 5A). The IGF-1 levels in the HepG2+*E*.*g*+Ghr^+^ (600 ng/mL, 8 h) group and the HepG2+*E*.*g*+Ghr^-^ (400 ng/mL, 8 h) group also had significant changes (*P* < 0.0001), (Fig 5B). However, exogenous alteration of the Ghrelin level in *in vitro* culture could not positively regulate IGF-1 secretion. We selected the HepG2 group, the HepG2+*E*.*g* group, the HepG2+*E*.*g*+Ghr^+^ (600 ng/mL, 8 h) group and the HepG2+*E*.*g*+Ghr^-^ (400 ng/mL, 8 h) group for comparative analysis. Supernatant ELISA showed that the MAO levels were significantly lower in the HepG2+*E*.*g*+Ghr^+^ (600 ng/mL, 8 h) group than in the other three groups (*P* = 0.0004), and the TGF-β1 levels were significantly lower in the HepG2+*E*.*g*+Ghr^+^ (600 ng/mL, 8 h) group than in the HepG2+*E*.*g* group and the HepG2+*E*.*g*+Ghr^-^ (400 ng/mL, 8 h) group (*P* < 0.0001). The MAO and PH levels were significantly higher in the HepG2 group than in the other three groups (*P* = 0.0004, *P* = 0.0025, respectively), and the TGF-β1 levels were significantly lower than in the other three groups (*P* < 0.0001), (Fig 5C and 5E). Cell extract WB showed that the relative protein levels of Smad3 were lower in the HepG2 group than in the HepG2+*E*.*g* group and the HepG2+*E*.*g*+Ghr^-^ (400 ng/mL, 8 h) group but higher than in the HepG2+*E*.*g*+Ghr^+^ (600 ng/mL, 8 h) group, however, the results were not significantly different (*P* = 0.8734), (Fig 5F and 5G). These data showed that *E*.*g* infection could activate the TGF-β1/Smad3 signaling pathway, but survival of the parasite may require fibrosis to maintain low expression. High expression of Ghrelin is able to inhibit the TGF-β1/Smad3 signaling pathway and mitigate fibrosis. In addition, cell extract WB showed that the relative protein levels of Myd88 in the HepG2+*E*.*g*+Ghr^+^ (600 ng/mL, 8 h) group were lower than the other 3 groups and significantly lower than those in the HepG2 group and the HepG2+*E*.*g* group (*P* = 0.0004), and the relative protein levels of NF-κB p65 in the HepG2+*E*.*g*+Ghr^+^ (600 ng/mL, 8 h) group were lower than those in the HepG2+*E*.*g* group and the HepG2+*E*.*g*+Ghr^-^ (400 ng/mL, 8 h) groups, the relative protein levels of TLR4 in the HepG2+*E*.*g*+Ghr^+^ (600 ng/mL, 8 h) group were lower than the other three groups, and the relative protein levels of Cyclin D1 and Cyclin E1 in the HepG2+*E*.*g*+Ghr^+^ (600 ng/mL, 8 h) group were higher than the other three groups, but due to the sample size, none of them were significantly different. cGAS and Sting showed no difference in the relative protein levels between groups, (Fig 5F, 5H and 5N). These data showed that *E*.*g* infection could activate the Myd88/NF-κB signaling pathway and may be mediated through TLR4 rather than cGAS/Sting. Highly expressed Ghrelin could inhibit the Myd88/NF-κB signaling pathway to reduce inflammation and promote liver proliferation. In addition, supernatant ELISA showed that the levels of Th1-type cytokine IL-2 was significantly lower in the HepG2+*E*.*g*+Ghr^+^ (600 ng/mL, 8 h) group than in the HepG2+*E*.*g* group and the HepG2+*E*.*g*+Ghr^-^ (400 ng/mL, 8 h) group (*P* < 0.0001), and the levels of IFN-γ and TNF-α were significantly lower than the other three groups (*P* < 0.0001), (Fig 5O). The levels of Th2-type cytokines IL-4 and IL-10 were significantly higher than the other three groups (*P* < 0.0001, *P* < 0.0001, respectively), (Fig 5P). Among them, the Th2-type cytokine IL-6, which is a pro-inflammatory factor, had the same changes as Th1-type cytokines and was significantly lower than the other three groups (*P* < 0.0001), (Fig 5P). In addition, the HepG2+*E*.*g*+Ghr^-^ (400 ng/mL, 8 h) group was able to significantly promote the secretion of TGF-β1 and IL-2 and inhibit the secretion of IL-10, exerting an effect opposite to that of Ghrelin compared to the HepG2+*E*.*g* group, (Fig 5E, 5O and 5P).

### Blockade of Ghrelin receptor could reduce PSCs survival rate in *in vitro* cultures

**Fig 6:**
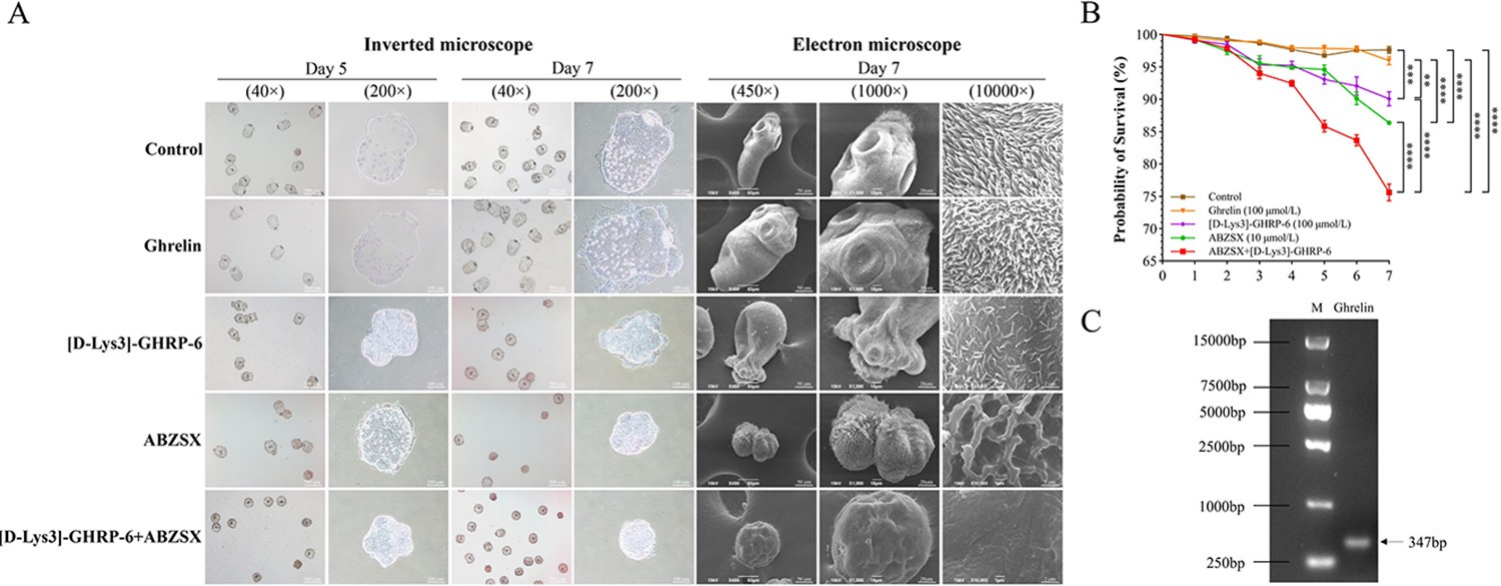
Effect of blocking the Ghrelin receptor on the survival rate of PSCs *in vitro* culture. (A) showed the observed images of light microscopy and scanning electron microscopy after intervention with different drugs in PSCs *in vitro* culture. (B) showed the survival rate analysis of PSCs *in vitro* culture. (C) showed the presence of gene expression of Ghrelin in PSCs by PCR. Data was considered statistically significant: * *P* < 0.05; ** *P* < 0.01; *** *P* < 0.001; **** *P* < 0.0001. PSCs: protoscoleces. ABZSX: Albendazole sulfoxide.

We cultured PSCs *in vitro* for 7 days and observed them under an inverted microscope. On day 5, the PSCs of the control group and Ghrelin group were transparent and oval in shape, and the hooks and suckers in the head segment and calcium granules in the body segment were evenly distributed and clearly visible, and the activity was obvious. On day 7, the volume of both groups increased compared with that on day 5, and the increase in the Ghrelin group was more obvious than that of the control group, but there was no significant difference. The survival rate of PSCs on day 7 was (97.62 ± 0.905) % for the control group and (95.96 ± 1.079) % for the Ghrelin group, with no significant difference. PSCs development was inhibited in the [D-Lys3]-GHRP-6 group, with a smaller size on day 5. Hooks were still visible in the cephalic segment, but some cephalic suckers and body calcium granules disappeared, and overall activity was slow. On day 7, some PSCs underwent atrophy and rupture, and died of tissue fluid extravasation, with a survival rate of (90.06 ± 1.902) %, which was significantly lower compared with the control group and the Ghrelin group (*P* = 0.0012). Comparing the ABZSX group with the [D-Lys3]-GHRP-6 group, the inhibition of the development of some PSCs was more obvious, and the hooks and suckers disappeared in the head node and calcium granules were visible in the body but distributed in a disordered way, the overall activity was slow, and some of them atrophied and died, and atrophy and death were more obvious on the 7th day, and the survival rate was (86.37 ± 0.529) %. The PSCs development of the ABZSX+[D-Lys3]-GHRP-6 group was severely inhibited, and some PSCs head nodal hooks and suckers and body calcium granules were lost. The survival rate at day 7 was (75.60 ± 2.225) %, which was significantly lower than the other groups (*P* < 0.0001), (Fig 6A and 6B). We performed electron microscopy scanning of PSCs on day 7, and the results showed that the PSCs of the control group and Ghrelin group were structurally normal, with the head nodes exposed, hooks and suckers clearly visible, and the microvilli were full and evenly distributed. In the [D-Lys3]-GHRP-6 group, the PSCs were smaller in size, and the hooks and suckers of some PSCs were still visible, but the neck and body were atrophied and sunken, with rupture and tissue fluid spillage, and the microvilli were sparse and unevenly distributed. The PSCs of the ABZSX group were atrophied and sunken severely, with the disappearance of the normal structure, and the head nodes, hooks and suckers could not be recognized, and the microvilli were sparse and stiff. The PSCs of the ABZSX+[D-Lys3]-GHRP-6 group were severely atrophied into a spherical shape, and all normal structures were unrecognizable, and the microvilli were close to completely disappearing, (Fig 6A). In addition, we detected the presence of Ghrelin gene expression in PSCs by PCR, (Fig 6C).

## Discussion

Previous studies had identified innate immune pathways such as inflammatory vesicles and Toll-like receptors activation, and hepatocyte apoptosis as the primary line of host defense against CE [30,64,65]. Recently, an increasing number of studies have found that growth metabolic pathways are activated during the progression of hepatic *Echinococcosis* infection and interact with immune-inflammatory and fibrotic pathways to co-regulate disease regression [26,66–71]. However, to date, few studies have addressed the various outcomes that result from interventions on the growth metabolic pathway during the progression of CE. Whether Ghrelin, as an important regulator of the growth metabolic pathway, is involved in regulating the progression of *E*.*g*-infected liver lesions has not been reported.

In the present study, we found that the serum Ghrelin levels were significantly reduced in *E*.*g*-infected mice during the late stage of infection and showed a significant negative regulatory relationship with liver GHSR. However, the serum Ghrelin levels were significantly elevated in *E*.*g*-infected mice during the early stage of infection, and protein expression of Ghrelin and GHSR was also significantly elevated in and around liver lesions. These results indicated that Ghrelin may be involved in regulating disease progression at least in the early stage of *E*.*g* infection.

Th1-type cellular immunity [6–12], Myd88/NF-κB [19,26] and TGF-β1/Smad3 [20,27–29] signaling pathways are activated to exert protective effects against parasite infection in the early stage of *E*.*g* infection. Studies had reported that Ghrelin could inhibit Th1-type cellular immunity and promotes Th2-type cellular immunity to exert an anti-inflammatory effect in liver fibrosis, cirrhosis, and liver injury diseases [39–43], and that it could inhibits NF-κB [44–47] and TGF-β1/Smad3 [45,48] signaling pathways through an interaction that reduces the secretion of pro-inflammatory factors and inhibits the proliferation and activation of HSCs to restore the dynamic balance of MMP2 and TIMP1, reduces the secretion of fibrogenic factor α-SMA, collagen I and III, and ameliorates chronic inflammation and fibrosis in the liver. However, this effect of Ghrelin may mediate disease progression in *E*.*g* infection. Our study revealed that compared to normal liver tissue, the expression of Ghrelin and receptor GHSR, Myd88/NF-κB and TGF-β1/Smad3 pathway proteins were concurrently increased in the liver lesions and peripheral inflammatory cell bands of *E*.*g*-infected mice. In addition, the mIHC results showed the expression of Ghrelin has an extremely strong co-localization with the expression of HSCs, T cells and TGF-β1/Smad3 pathway proteins, and a strong co-localization with the expression of macrophages, Myd88/NF-κB pathway proteins. These results indicated that Ghrelin is highly expressed in the early stage of *E*.*g* infection and may be involved in suppressing the expression of hepatic immunoinflammation and fibrosis mainly by acting on HSCs and T cells to mediate the progression of hepatic infectious lesions. In addition, we also observed the presence of protein expression of Ghrelin on the inflammatory cell nuclei and lysosomes by IEM, showing a correlation between Ghrelin and the regulation of immunoinflammation and autophagy.

In this study, we confirmed that PSCs proteins were able to concentration-dependently stimulate the up-regulation of Ghrelin secretion in *in vitro* culture of HepG2 cells. We further used *in vitro* co-culture experiments with HepG2 cells and PSCs proteins and found that compared to single HepG2 cells culture, exogenous Ghrelin protein administration was able to inhibit the secretion of pro-inflammatory factors IL-2, INF-γ, TNF-α, IL-6, Myd88, NF-κB p65 and fibrogenic factors MAO, TGF-β1, Smad3, and promote the secretion of anti-inflammatory factors IL-4 and IL-10. The administration of the Ghrelin receptor blocker [D-Lys3]-GHRP-6 increased the secretion of the pro-inflammatory factor IL-2 and the fibrotic factor TGF-β1 and decreased the secretion of the anti-inflammatory factor IL-10. The results of *in vitro* experiments indicated that high Ghrelin expression during the course of *E*.*g* infection could suppress Th1-type cellular immunity, the Myd88/NF-κB and TGF-β1/Smad3 signaling pathways to exert inhibitory immunoinflammatory and fibrotic effects. Inhibition of these two signaling pathways in turn was able to directly inhibit fibrogenesis mediated by proliferative activation of HSCs [49]. Ghrelin is able to promote the secretion of cell cyclin protein to ameliorate hepatic injury [49], and *in vitro* experiments had also shown that Ghrelin promotes the secretion of the proliferative factors Cyclin D1 and Cyclin E1, which may further ameliorate the hepatic injury induced by *E*.*g* infections. However, T-cell immune activation and proliferative activation of HSCs are important host barriers against parasite infection, and these effects of Ghrelin favor the occurrence of immune escape and tolerance by the parasite and promote the progression of *E*.*g* infection. *In vitro* culture drug intervention experiments in PSCs supported this conclusion. Inhibition of the Ghrelin receptor by [D-Lys3]-GHRP-6 could significantly slow PSCs growth, reduce PSCs survival rate and improve the insecticidal efficacy of ABZSX. Our previous studies had reported the presence of an intact insulin signaling pathway and a TGF-β/Smad signaling pathway in PSCs [72–75], and Ghrelin has been shown to be an important regulator of both signaling pathways [49], which could partially explain the results of blocking the Ghrelin receptor to reduce PSCs survival rate. Interestingly, we detected gene expression of Ghrelin in PSCs, which indicated that its growth and metabolism is Ghrelin-dependent.

Ghrelin could regulate hepatic IGF-1 secretion via the "gastrointestinal-brain-liver axis" [49–51]. Studies have reported that IGF-1 could accelerate the progression of parasitic infectious diseases [55–59]. Our previous study played a role against *E*.*g* infection by blocking IGF-1R was able to interfere with glycolipid metabolism of PSCs leading to vesicle collapse [76]. The results of the present study showed a significant reduction in the serum IGF-1 levels positively regulated by Ghrelin during the late stage of infection in *E*.*g*-infected mice. However, the serum IGF-1 levels were significantly elevated in the early stage of infection in *E*.*g*-infected mice and were significantly positively correlated with Ghrelin. The protein expression of IGF-1 in and around liver lesions was also significantly elevated. These results indicated that Ghrelin may also mediate disease progression by regulating IGF-I secretion in the early stage of *E*.*g* infection. Interestingly, we found that PSCs protein stimulation of HepG2 cells was able to upregulate both Ghrelin and IGF-1 secretion with a significant positive correlation in *in vitro* experiments. However, exogenous Ghrelin administration could not positively correlate with regulating IGF-1 secretion. This finding showed that the positive regulation of IGF-1 by Ghrelin is "gastrointestinal-brain-hepatic axis" and vagal system-dependent, and could not be directly regulated. This is in line with previous findings [77], and the direct regulatory relationship between Ghrelin and IGF-1 is a hot issue to be revealed [37].

**Fig 7:**
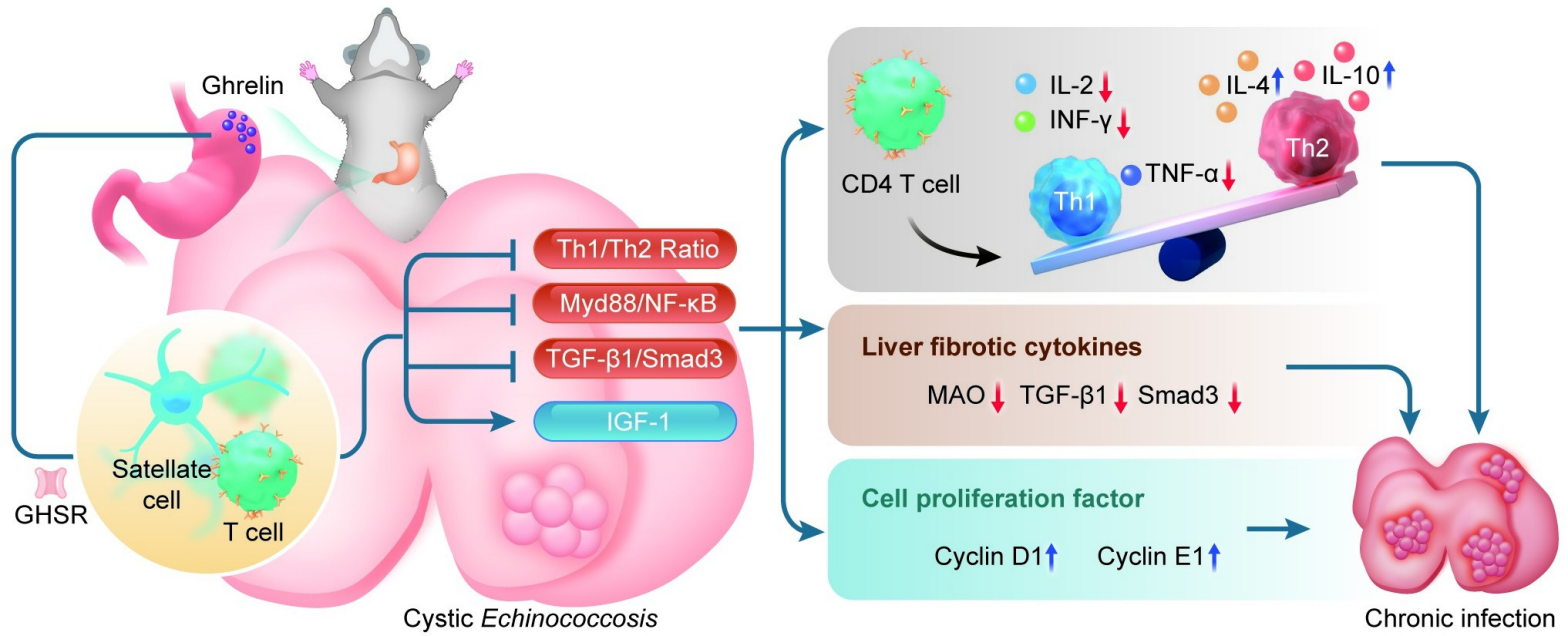
Ghrelin could modulate the progression of liver lesions in the early stages of hepatic *E*.*g* infection by suppressing immunoinflammation and fibrosis in mice. The picture showed that gastric fundus-secreted Ghrelin combined with GHSR is able to act on T cells and HSCs to inhibit Th1-type cellular immunity, Myd88/NF-κB and TGF-β1/Smad3 signaling pathways, and regulate hepatic secretion of IGF-1, which up-regulates the secretion of the anti-inflammatory factors IL-4 and IL-10, down-regulates the secretion of the pro-inflammatory factors IL-2, INF-γ, TNF-α and the fibrogenic factors MAO, TGF-β1, Smad3, and promote the cell proliferation factors Cyclin D1 and Cyclin E1 secretion, which together mediate the progression of *E*.*g*-infected liver lesions. *E*.*g*: *Echinococcus granulosus*. HSCs: hepatic stellate cells. MAO: monoamine oxidase. The author used Adobe Illustrator to hand-draw the images, and the original images had no copyright dispute.

In conclusion, we found some evidence that Ghrelin is involved in regulating the progression of liver lesions in the early stages of hepatic *E*.*g* infection in this study. Preliminarily, we revealed that Ghrelin is highly expressed in the early stage of hepatic *E*.*g* infection and may act mainly on HSCs and T cells, exerting biological effects to inhibit the TGF-β1/Smad3 and Myd88/NF-κB signaling pathways, as well as mediating the conversion of T-cell immunity to the Th2 type, which in turn suppresses the expression of immunoinflammation and fibrosis, combined with modulation of hepatic IGF-1 secretion, jointly mediate the progression of hepatic infectious lesions, (Fig 7A). Blocking Ghrelin receptor has been shown to significantly inhibit PSCs growth, at least *in vitro*. Further studies in *in vivo* intervention experiments could help to provide insight into the mechanisms by which Ghrelin regulates hepatic *E*.*g* infection and provide potential targets for drug therapy.

## Supporting information

S1 Data The raw data of this study. (XLSX)
